# Localized T3 production modifies the transcriptome and promotes the hepatocyte-like lineage in iPSC-derived hepatic organoids

**DOI:** 10.1172/jci.insight.173780

**Published:** 2023-12-08

**Authors:** Jorge Hidalgo-Álvarez, Federico Salas-Lucia, Diana Vera Cruz, Tatiana L. Fonseca, Antonio C. Bianco

**Affiliations:** 1 Section of Adult and Pediatric Endocrinology, Diabetes and Metabolism, and; 2 Center for Research Informatics, The University of Chicago, Chicago, Illinois, USA.

**Keywords:** Endocrinology, Metabolism, Embryonic development, Thyroid disease, iPS cells

## Abstract

Thyroid hormone (TH) levels are low during development, and the deiodinases control TH signaling through tissue-specific activation or inactivation of TH. Here, we studied human induced pluripotent stem cell–derived (iPSC-derived) hepatic organoids and identified a robust induction of *DIO2* expression (the deiodinase that activates T4 to T3) that occurs in hepatoblasts. The surge in DIO2-T3 (the deiodinase that activates thyroxine [T4] to triiodothyronine [T3]) persists until the hepatoblasts differentiate into hepatocyte- or cholangiocyte-like cells, neither of which expresses *DIO2*. Preventing the induction of the DIO2-T3 signaling modified the expression of key transcription factors, decreased the number of hepatocyte-like cells by ~60%, and increased the number of cholangiocyte-like cells by ~55% without affecting the growth or the size of the mature liver organoid. Physiological levels of T3 could not fully restore the transition from hepatoblasts to mature cells. This indicates that the timed surge in DIO2-T3 signaling critically determines the fate of developing human hepatoblasts and the transcriptome of the maturing hepatocytes, with physiological and clinical implications for how the liver handles energy substrates.

## Graphical abstract

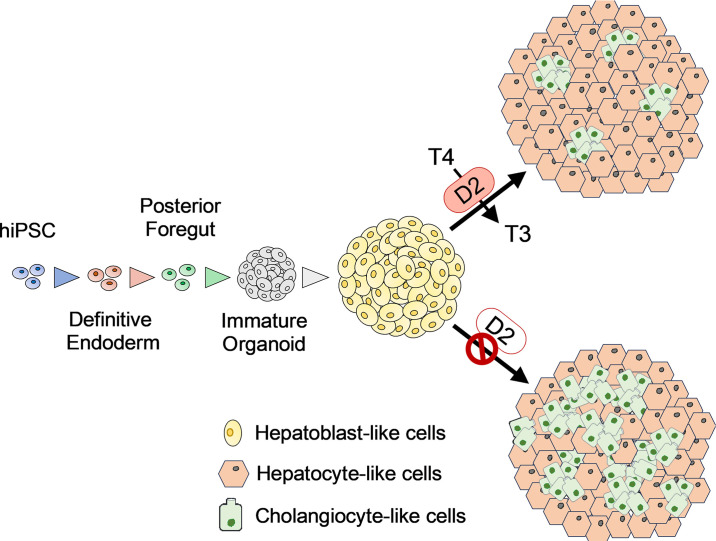

## Introduction

Thyroid hormones (THs) play important metabolic functions in all vertebrates, with the liver being one of their primary targets. THs accelerate hepatic glucose output, the synthesis of fatty acids and ketogenesis, and multiple steps in the metabolism of cholesterol, predominantly through interactions with the TH receptor β (TRβ) (1–3). The metabolic effects of TH in the liver can be traced back to the development of hepatocytes when there is an acute surge in TH signaling shortly after birth due to a transient peak of *Dio2* expression (4). *Dio2* encodes the type 2 deiodinase (D2), which locally activates the prohormone thyroxine (T4) to the biologically active T3. This programmed expression of *Dio2* is not unique to the liver, being observed in the developing bone (5), skeletal muscle (6, 7), brown adipose tissue (8), and retina (9), with the peak of D2 activity occurring as late as P15, as observed in the cochlea (10). This mechanism ensures that the developing cells are exposed to a pulse of TH signaling at a predefined critical moment during development (11–13).

The transient surge of *Dio2* in the liver occurs during P1 and P2, and it is so effective that it doubles the local T3 concentration and TH signaling. Mice with hepatocyte-specific *Dio2* inactivation exhibit a delay in neonatal hepatic expression of key lipid-related genes and a persistent reduction in *Pparγ* expression (4). Even though *Dio2* is only expressed in the perinatal liver, *Dio2* inactivation results in a significant phenotype in adult mice. Adult mice with liver-specific *Dio2* inactivation exhibit markedly different responses to 2 common insults to the liver — i.e., high-fat diet or chronic and binge ethanol feeding (4, 14). Whereas control mice develop steatosis in response to both conditions, this is not observed in mice with liver-specific *Dio2* inactivation. These mice do not exhibit the typical induction of hundreds of genes involved in the synthesis of fatty acids and triglycerides. Their hepatic response involves a different set of genes associated with lipid transport and export, reverse cholesterol transport, and lipase activity (4, 14).

A corollary for these studies is that the programmed perinatal pulse of *Dio2* and T3 signaling observed in the developing liver results in an adult liver with a substantial shift in the way it handles lipogenic stimuli and with an excess of fat, favoring fat accumulation in the liver (4, 14). While this might seem a maladaptive response, since it favors fat deposition and liver steatosis, from an evolutionary point of view, this is probably adaptive and advantageous, given that the limited availability of food most likely served to select animals with greater ability to preserve energy substrates.

Subsequent studies revealed that the perinatal D2-T3 pulse acts by transiently reducing the levels of H3K9me3 and eliminating about 1,500 discrete DNA methylation sites (15). This increases chromatin accessibility and the expression of hundreds of genes involved in broad aspects of hepatocyte function, including lipid metabolism. Along with *Pparγ*, the expression of zinc-finger protein-125 (*Zfp125*) in the adult mouse liver is also increased by the perinatal pulse of D2-T3. Zfp125 is a Foxo1-inducible transcriptional repressor that acts by repressing genes involved in lipoprotein structure, lipid binding, and transport and causes lipid accumulation by reducing the liver secretion of triglycerides and hepatocyte efflux of cholesterol (16).

To investigate the potential role of *DIO2* expression in the development of the human liver and whether the D2-T3 triggers similar mechanisms that are at play in the mouse liver, here we used 3D hepatic organoids derived from human induced pluripotent stem cells (iPSCs) and discovered that the pulse of *DIO2* expression starts at the early stages of hepatoblast formation and lasts through the transition to more mature cells. The resulting pulse of locally produced T3 affects the expression of key transcription factors and the ultimate fate of the differentiating hepatoblasts as well as the transcriptome of the maturing hepatocytes.

## Results

In humans, the organization of the endoderm and posterior foregut (PFG) occurs during the first 2–3 weeks (E7–E8.5 in mice). Subsequently, liver organogenesis starts at week 4, and the liver bud is created, where the hepatoblast migration and proliferation take place between weeks 5 and 7 (17, 18) (E9–E14 in mice; refs. 17, 19). Lastly, hepatoblasts differentiate into hepatocytes or cholangiocytes during weeks 7–30 (E13.5–E18.5 in mice). Here, we studied the developmental role played by *DIO2* by using human iPSCs to generate hepatic organoids, which exhibit developmental stages that resemble those in human hepatic organogenesis.

### Differentiation of iPSCs into hepatic organoids

**Figure 1:**
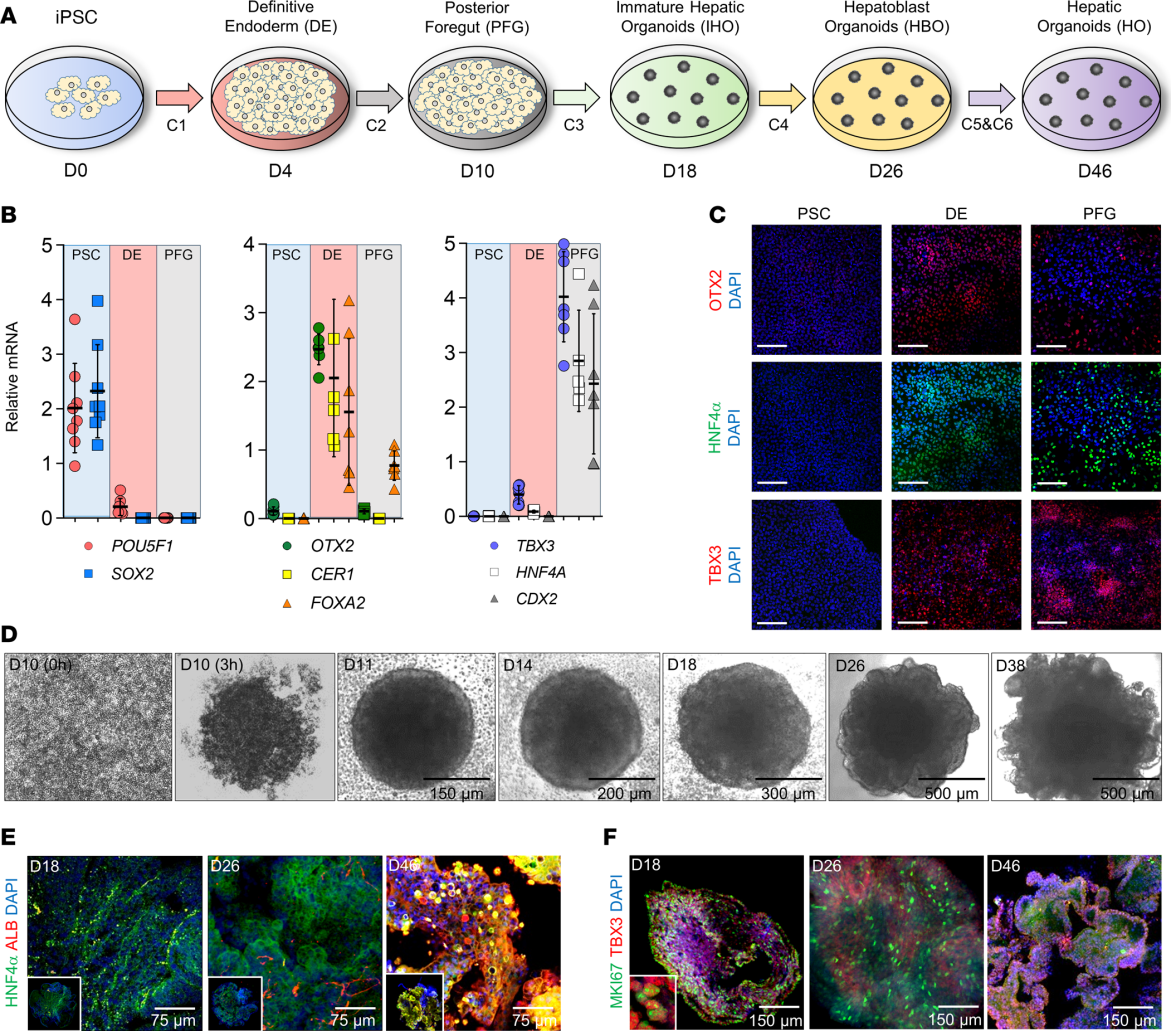
Differentiation of human iPSC into hepatic organoids. (**A**) Graphic representation of the differentiation phases. The first phase involves iPSC (day 0), DE (day 4), and PFG (day 10) in a 2-dimensional culture, and the second phase comprises IHO (day 14 to day 18), HBO (day 22 to day 26), and HO, which differentiated into a 3-dimensional structure since day 10. The maturation of the HO encompasses 2 phases, from day 27 to day 38 (HO1) and the second from day 39 to day 46 (HO2). C1–C6 are the indicated differentiation cocktails used (see Methods). (**B**) Relative mRNA levels of iPSC (*POU5F1*, *SOX2*), DE (*OTX2*, *CER1*, *FOXA2*), and PFG (*TBX3*, *HNF4A*, *CDX2*) markers; β*-*actin was used as the internal control. Entries are the mean of duplicates, represented as aligned scatter dot plots and the mean ± SD (iPSC, *n* = 8; DE and PFG *n* = 7; *HNF4A* in PFG, *n* = 6), and each differentiation stage is indicated at the bottom of the graphs. (**C**) Immunofluorescence of OTX2 (red), HNF4α (green), and TBX3 (red) in iPSC, DE, and PFG cells; nuclei are in blue. Scale bar: 150 μm. (**D**) Representative bright-field images of self-organized hepatic organoids from day 10 to day 38. (**E**) Immunofluorescence of HNF4α (green) and albumin (red) at day 18, day 26, and day 46. (**F**) Immunofluorescence of the proliferative markers MKI67 (green) and TBX3 (red) at day 18, day 26, and day 46. Nuclei are shown in blue; the inset is magnified on the bottom left of the first panel.

To monitor the progression of the differentiating iPSCs into hepatic organoids, we tracked the expression of the stage-specific cell markers over time (Figure 1, A–F). As expected, the expression of the iPSC markers (20) *POU5F1* (also known as *OCT4*) and *SOX2* decreased after the differentiation into definitive endoderm (DE), at the same time that the DE markers *OTX2*, *CER1*, and *FOXA2* increased (Figure 1B). Subsequently, the increase in the mRNA levels of *HNF4A*, *TBX3*, and *CDX2* (21, 22) signaled the organization of the PFG (Figure 1B). The changes in the expression of these markers (OTX2, HNF4α, and TBX3; ref. 23) were also tracked through immunofluorescence (Figure 1C).

On day 10, the 2D cultures were disrupted by dissociation, and subsequently, the cells self-reestablished into a 3D structure that grew progressively under stimulation of key reagents in the differentiation cocktails — i.e., BMP4, BMP7, FGF7, and the WNT agonist CHIR99021 — to promote the hepatic specification (Figure 1D) (17). This occurred during the transition into 3D organoids and started with immature hepatic organoids, followed by hepatoblast organoids, and finally hepatic organoids (Figure 1A). These transitions were monitored by immunofluorescence of the developmental markers HNF4α (Figure 1E) and TBX3 (Figure 1F), together with the hepatocyte marker albumin (Figure 1E) and the proliferative marker MKI67 (Figure 1F).

**Figure 2:**
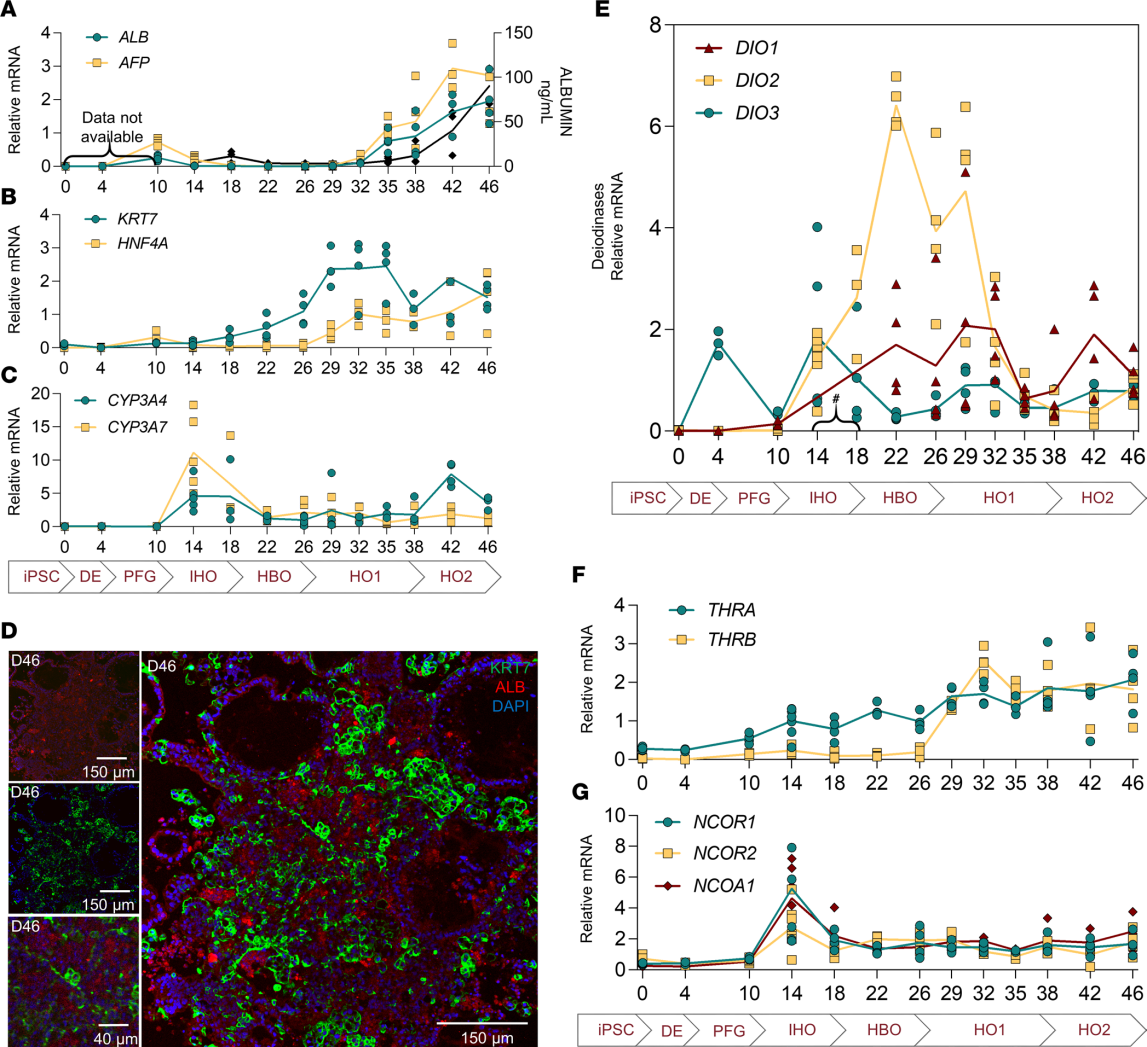
Gene markers and deiodinase expression patterns during the process of differentiation into hepatic organoids. (**A**) Relative mRNA levels of *ALB* and *AFP* (left *y* axis) and albumin levels in the medium (right *y* axis). Albumin levels in the medium from day 14 to day 46; for day 14 and day 18, each sample was obtained from 10 individual wells and combined; for day 22 to day 46, there were 10 organoids per well (*n* = 2, day 14, day 22, day 46; *n* = 3, D18 and day 26; *n* = 4, day 29, day 42; *n* = 5, day 32, day 38; *n* = 6, day 35). (**B**) Relative mRNA levels of *KRT7* and *HNF4A*. (**C**) Relative mRNA levels of cytochrome P450 isoforms, *CYP3A7* (fetal liver marker) and *CYP3A4* (adult liver marker). (**D**) Immunofluorescence at day 46 of albumin (hepatocyte marker; red; top left; scale bar: 150 μm) and KRT7 (cholangiocyte marker; green; middle left; scale bar: 150 μm); the inset is magnified (bottom left; scale bar: 40 μm), and the merge of both images (right). Nuclei are shown in blue. (**E**) Relative mRNA levels of *DIO1*, *DIO2*, and *DIO3*. (**F**) Relative mRNA levels of nuclear thyroid receptor, *THRA* and *THRB*. (**G**) Relative mRNA levels of nuclear coactivator *NCOA1* and corepressors *NCOR1* and *NCOR2*. β*-*Actin was used as the internal control; all entries are the mean of duplicates and shown as aligned scatter dot plots (mRNA samples: day 4, *n* = 3; day 14, *n* = 7; rest, *n* = 4). ^#^Data not available for *DIO1*. The days of the differentiation are shown on the *x* axis (see Figure 1).

The presence of maturing hepatocytes was detected through *HNF4A* and albumin (*ALB*) mRNA and by human albumin in the conditioned medium (Figure 2, A and B). Furthermore, HNF4α and albumin^+^ cells were visualized on day 46 organoids (Figure 1E). *AFP* is highly expressed by the embryonic hepatocytes in the fetal liver. Here, *AFP* mRNA levels were briefly detected on day 10 and increased progressively to reach an ~3-fold increase by day 42 (Figure 2A). In addition, we also measured mRNA for the hepatocyte genes *CYP3A7*, a major fetal hepatic enzyme, and *CYP3A4*, a cytochrome P450 isoform involved in the drug metabolism in adults (Figure 2C). *CYP3A7* expression increased at day 14 by ~10-fold; it then diminished and remained steady (~2-fold) until day 46 (Figure 2C). A similar pattern was observed for *CYP3A4* except that, at day 42, there was an ~7-fold increase in expression (Figure 2C). At the same time, *KRT7* mRNA, a typical cholangiocyte marker, appeared on day 22 (Figure 2B), increased by ~3-fold, and reached a plateau from day 29 to day 35 (Figure 2B). These results indicate that the differentiation of iPSCs into hepatic organoids generates developing hepatocyte-like and cholangiocyte-like cells (Figure 2D).

### A transient surge in DIO2 expression during hepatoblast differentiation

We previously identified *Dio2* expression in E13.5–E18.5 mouse liver embryos (4), a timing during which there is differentiation of hepatoblasts into hepatocytes (17). Here we also identified a peak of DIO2 during the differentiation of hepatoblasts into hepatocytes. *DIO2* mRNA surged on day 14 to reach an ~6-fold expression peak by day 22 that was sustained until day 29 (Figure 2E) and subsequently subsided (Figure 2E). D2 catalytic activity reached ~0.35 nmol T3/h/mg protein on day 22 and dropped by half on day 38 (Supplemental Figure 1A; supplemental material available online with this article; https://doi.org/10.1172/jci.insight.173780DS1). At the same time, *DIO3* expression (the enzyme that inactivates TH) remained low throughout the differentiation process, except for day 4 and day 14, when a subtle but short-lived elevation was observed (Figure 2E and Supplemental Figure 1B). *DIO1* mRNA emerged to ~2-fold at day 22 until day 32 (Figure 2E). It then increased again ~2-fold at day 38 (Figure 2E). *THRA* mRNA levels were low during the early stages of differentiation, but after day 10, *THRA* mRNA levels increased progressively to reach ~2-fold by day 46 (Figure 2F). *THRB* expression was induced after day 26 by about 2-fold and remained high through day 46 (Figure 2F), along with a steady presence of the TR coregulators *NCOR1*, *NCOR2*, and *NCOA1* (also known as *SRC1*) (Figure 2G).

### Local D2-mediated T3 production affects the differentiation of hepatoblasts

**Figure 3:**
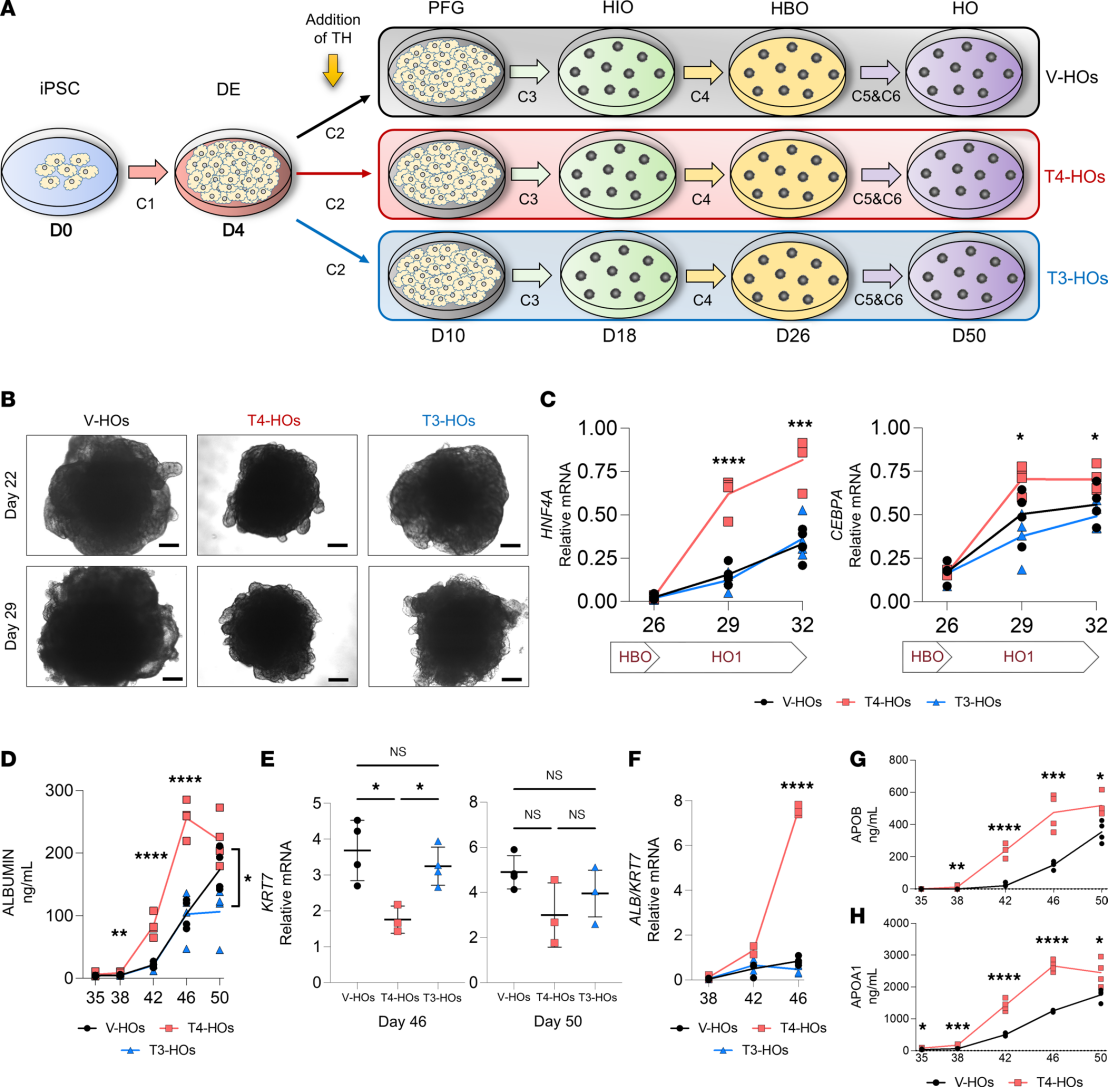
Formation of hepatic organoids in the presence or absence of TH. (**A**) Graphic representation of the development of hepatic organoids in the presence or absence of TH. The yellow arrow indicates the addition of TH (from day 5 to day 50). The final concentration of T4-HOs (red) and T3-HOs (blue) was 1 nM T4 (free T4 = ~15 pM) and 200 pM T3 (free T3 = ~10 pM); V-HOs were grown in the absence of T4 or T3 (black). C1–C6 are the indicated differentiation cocktails used (see methods). (**B**) Bright-field images (3 conditions) of hepatoblast at day 22 and hepatic organoids at day 29. Scale bar: 200 μm. (**C**) Relative mRNA levels of *HNF4A* (hepatocyte marker) and *CEBPA* from day 26 to day 32 (*n* = 4). (**D**) Albumin levels in the medium from day 35 to day 50 (*n* = 4). (**E**) Relative mRNA levels of *KRT7* at day 46 and day 50 (*n* = 4, except T4-HOs, *n* = 3). (**F**) *ALB*/*KRT7* mRNA ratio during from day 38 to day 46 (*n* = 4 except T4-HOs and T3-HOs at day 42, *n* = 2; T4-HOs at day 46, *n* = 3). (**G** and **H**) Apolipoprotein B (APOB) and Apolipoprotein A1 (APOA1) levels from the medium from day 35 to day 50, comparing V-HOs (black) versus T4-HOs (red). Ten organoids per well (*n* = 4). Two-tailed Student’s *t* test for comparing V-HOs versus T4-HOs, and 1-way ANOVA and Tukey test were used for multiple comparisons. Data are the mean of duplicates, represented as aligned scatter dot plots and their mean. *P < 0.05; **P < 0.01; ***P < 0.001; ****P < 0.0001. The days of the differentiation are shown on the *x* axis (see legend Figure 1).

The D2 pathway is only active if T4 is available, whether availability is from plasma or supplied in the medium. The differentiation cocktails (C2–C6) do not contain T4, but B27 contains T3 at above the physiological levels (24). Thus, to eliminate the T3 signaling and the local D2-mediated T4-to-T3 activation during the development of liver organoids, we cultured DE cells (day 4) in B26 (without T3) medium containing no TH (vehicle-only [V-HOs]) or containing only ~10 pM free T3 (T3-HOs), which is equivalent to physiological plasma T3 concentration. The results were contrasted with cells grown with ~15 pM free T4 (T4-HOs), supporting physiological D2-mediated T3 production (Figure 3A).

Our first approach was to measure the volume of the developing organoids, which increased progressively from day 15 to day 38 (Supplemental Figure 1C). This is reflected in the ~2-fold–higher mRNA levels for the proliferative marker *MKI67* in day 22 as compared with day 10 (Supplemental Figure 1D). The growth of the organoids was affected by the functionality of the D2-generated T3 pathway. At day 22, T4-HOs were 20%–25% smaller than V-HOs and ~15% smaller than T3-HOs (Figure 3B and Supplemental Figure 1E). No differences were observed between T3-HOs and V-HOs (Supplemental Figure 1E). On day 29, both T4-HOs and T3-HOs were ~20% smaller than V-HOs (Figure 3B and Supplemental Figure 1E). Accordingly, the absence of T4 in the medium increased *MKI67* mRNA levels by ~20% and ~75% on day 14 and day 18, respectively (Supplemental Figure 1D). These differences confirmed our hypothesis that the local D2-mediated T3 production affects the differentiation process of hepatic organoids.

We next measured the expression of 2 TFs involved in hepatocyte differentiation, *HNF4A* and *CEBPA*, on day 26 to day 32 (Figure 3C), along with expression of 2 differentiation-specific markers, *ALB* and *KRT7*, during day 38 to day 50 (Figures 3, D–F, and Supplemental Figure 1F). First, we noticed that *HNF4A* and *CEBPA* mRNA levels began to increase at day 29, with *HNF4A* reaching higher levels in T4-HOs (Figure 3C). In addition, on day 42 and day 46, *ALB* mRNA levels remained substantially higher in the T4-HOs as compared with T3-HOs or V-HOs (Supplemental Figure 1E). These differences were also reflected in the albumin (Figure 3D), and apolipoprotein (*APO*) levels in the conditioned media of T4-HOs. Albumin levels were 3.0-fold and 2.5-fold higher than in V-HO on day 42 and day 46 (Figure 3D), respectively, whereas APOB levels were ~10-fold and 3-fold higher (Figure 3G) and APOA1 levels were ~3-fold and ~2-fold higher (Figure 3H) at day 42 and day 46, respectively. Conversely, *KRT7* mRNA levels on day 46 and day 50 were lower in T4-HOs but not in T3-HOs (Figure 3E). The magnitude of differences between *ALB* and *KRT7* expression during day 46 can be appreciated by plotting the ratio of *ALB/KRT7* (Figure 3F). These results suggest that the local D2-mediated T3 production favors the differentiation of the hepatoblasts toward hepatocytes versus cholangiocytes.

### The temporal expression of TFs is modified by local D2-mediated T3 production

**Figure 4:**
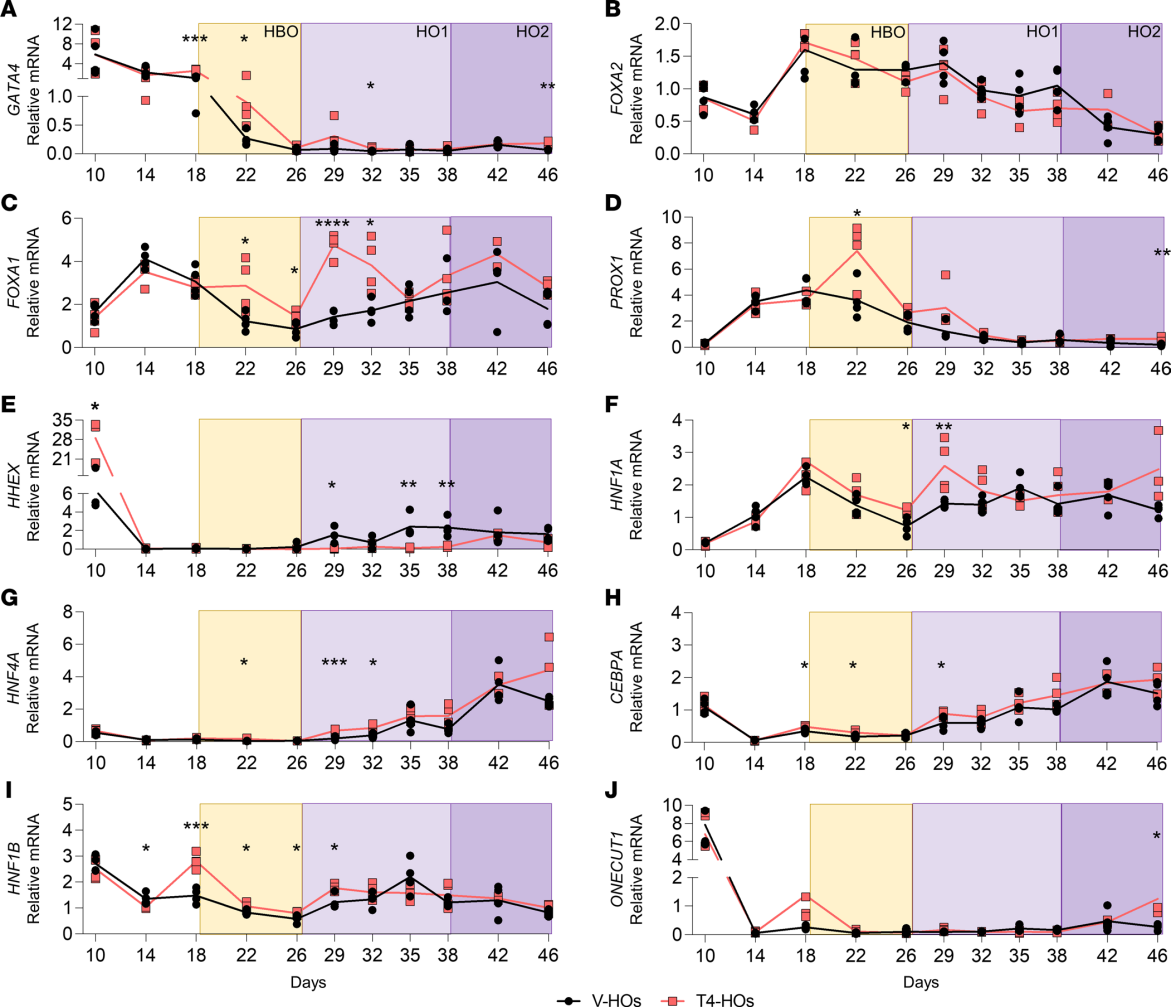
Expression of TFs involved in liver development. (**A**–**J**) Relative mRNA levels of TFs (*GATA4*, *FOXA2*, *FOXA1*, *PROX1*, *HHEX*, *HNF1A*, *HNF4A*, *CEBPA*, *HNF1B*, *ONECUT1*) involved in the network regulation of the hepatic organoids from day 10 to day 46 in V-HOs (black) and T4-HOs (red) (*n* = 4 except T4-HOs at day 42, *n* = 2; day 46, *n* = 3; T4-HOs at day 18 in *FOXA2*, *PROX1*, and at day 10 in *HHEX* *n* = 3; and V-HOs at D29 in *HHEX*, *n* = 3). β*-*Actin was used as the internal control. The yellow square represents the hepatoblast expansion (HBO), and the purple squares indicate the period of hepatocyte and cholangiocyte maturation (HO1-2). Two-tailed Student’s *t* test was used to compare groups each day. Data are the mean of duplicates and represented as aligned scatter dot plots. *P < 0.05; **P < 0.01; ***P < 0.001; ****P < 0.0001. The days of the differentiation are shown on the *x* axis (see Figure 1).

The local D2-T3 signaling also affected other key TFs, including the pioneer factors *GATA4* (role in liver bud expansion) and *FOXA1* (Figure 4, A–C). *GATA4* was highly expressed on day 10 to day 18 and decreased progressively during organoid maturation (Figure 4A). In cells capable of local D2-mediated T3 production, *GATA4* mRNA levels were ~2.5-fold higher on day 18 and ~3-fold higher on day 22 (Figure 4A). *FOXA2* mRNA levels were not modified by the presence of T4 (Figure 4B). In these cells, *FOXA1* mRNA peaked on day 14, decreased during the hepatoblast, and slowly increased again until organoids (Figure 4C). A functional D2-T3 pathway had a generalized positive effect on *FOXA1* expression, with ~130% and 70% higher mRNA levels on day 22 and day 26, respectively (Figure 4C); much higher fold inductions by D2-T3 were observed at day 29 and day 32 (respectively, ~3-fold and ~2-fold; Figure 4C).

*PROX1* promotes the migration and proliferation of the hepatoblast during the early liver bud formation. Its mRNA levels were low up until day 10, when it increased progressively to reach a plateau by day 18 and subsequently dropped over time (Figure 4D). In hepatoblasts and in early hepatic organoids, a functional D2-mediated T3 production increased the expression of *PROX1* by ~2-fold (day 22) (Figure 4D). *HHEX* is another early TF that plays a role in the formation of the liver bud and, later on, the development of the biliary tree. The *HHEX* mRNA levels were high by day 10 and decreased sharply by day 14 (Figure 4E). A functional D2-mediated T3 production mechanism increased this early expression peak of *HHEX* by ~3-fold but, during the later phases of development, markedly reduced *HHEX* expression by ~10-fold (Figure 4E).

The 3 hepatocyte-promoting TFs, *HNF1A*, *HNF4A* (which, together with *CEBPA* promotes albumin production and with *HNF1A* maintains and directs the hepatocyte fate; refs. 25, 26), and *CEBPA*, exhibited low expression levels in the early hepatoblasts but increased over time; by organoids, their expression levels had increased 2- to 4-fold (Figure 4, F–H). A functional D2-mediated T3 production mechanism had an overall stimulatory effect on these 3 TFs. During the different developmental periods, D2-T3 caused *HNF1A* mRNA levels to increase by ~2-fold (Figure 4F), *HNF4A* mRNA levels by 2- to 5-fold (Figure 4G), and *CEBPA* mRNA by 40%–75% (Figure 4H).

*HNF1B* and *ONECUT1* (also known as *HNF6*) are TFs that play a role in bile duct morphogenesis (Figure 4, I and J). *HNF1B* expression remained stable throughout development (Figure 4I), but *ONECUT1* mRNA levels peaked at day 10, sharply diminished to low levels by day 14, and remained low (Figure 4J). Here, the local D2-mediated T3 production stimulated *HNF1B* expression by 25% to 2-fold in hepatoblasts and early organoids (Figure 4I), whereas *ONECUT1* was potently induced by D2-T3 (~4.5- to 5-fold) exclusively at day 46 (Figure 4J).

### Hepatic organoids are made up of hepatocyte-like and cholangiocyte-like cells

**Figure 5:**
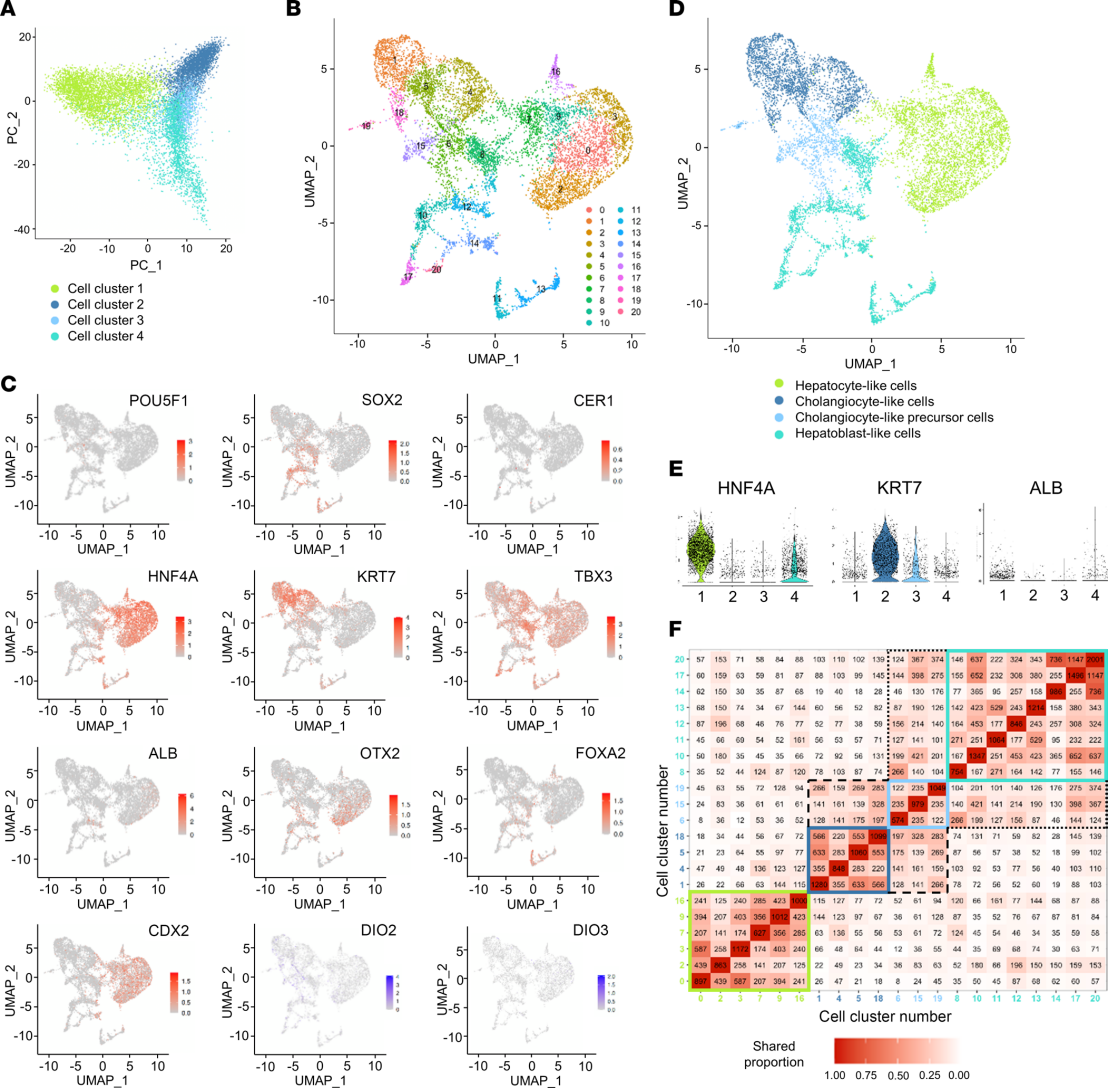
scRNA-Seq identification of cells in hepatic organoid at day 45. (**A**) PCA of the 2 first principal components (PC1 versus PC2) of hepatic organoids at day 45 (*n* = 10,887 cells). (**B**) UMAP visualization with a SSN at 0.8 resolution. (**C**) UMAP distribution plot of differentiation markers: *POU5F1* and *SOX2* (pluripotency markers); *CER1*, *OTX2*, and *FOXA2* (DE markers); *CDX2* and *TBX3* (PFG markers); *HNF4A* and *ALB* (hepatocyte markers); *KRT7* (cholangiocyte marker); and *DIO2* and *DIO3*. (**D**) UMAP visualization after grouping the clusters. (**E**) scRNA-Seq violin plots of markers *HNF4A*, *KRT7*, and *ALB* in the cell groups. 1 indicates hepatocyte-like cells; 2 indicates cholangiocyte-like cells; 3 indicates cholangiocyte-like precursor cells; and 4 indicates hepatoblast-like cells. (**F**) A plot of the number of conserved genes shared across the clusters. Colored squares (green, dark blue, sky blue, turquoise) indicate the cluster of cells that make up every group. Black dotted and dashed lines indicate a resemblance among groups.

Single-cell RNA-Seq (scRNA-Seq) was used to analyze a total of 10,887 day 45 cells (Figure 5A), which were sorted into 21 clusters (Figure 5B) distributed in 4 groups based on (a) the expression and cell positivity level of 10 developmental genes (Figure 5C and Supplemental Figure 2A), (b) the number of conserved genes expressed in each cluster (Figure 5F and Supplemental Table 1), and (c) the top 20 genes expressed in each cluster (Supplemental Table 2):

#### Hepatocyte-like cells.

This group consisted of 6 cell clusters (clusters 0, 2, 3, 7, 9, and 16) expressing *HNF4A* (26), *CDX2*, and the typical hepatocyte markers (i.e., *FGG*, *FGB*, *APOC3*, *APOA4*, and *GSTA1*) (Figure 5C, Supplemental Figure 2A, and Supplemental Table 1) as well as a high number of conserved genes (Figure 5F). 

#### Cholangiocyte-like cells.

Using a similar rationale, we identified 4 clusters (clusters 1, 4, 5, and 18), all of which expressed *KRT7*; other typical cholangiocytes markers — e.g., *KRT8*, *PROM1*, *SOX9*, and *HNF1*β — were not highly expressed (Supplemental Table 1).

#### Hepatoblast-like cells.

The next 8 most similar clusters (clusters 8, 10, 11, 12, 13, 14, 17, and 20) expressed the early developmental markers *SOX2* (iPSC), *HNF4A*, and *FOXA2* (DE, PFG) not present in the hepatocyte- or cholangiocyte-like groups of cells (Figure 5C). These clusters also expressed *OTX2* (DE), *CDX2* (PFG), and *ALB* (all expressed in hepatocyte-like cells but not in cholangiocyte-like cells; Figure 5, C–E).

#### Cholangiocyte-like precursor cells.

The last 3 unassigned clusters (clusters 6, 15, and 19) expressed *KRT7* and *SOX2* (Figure 5, C and E, and Supplemental Figure 2A). This profile suggested an intermediate group identity between hepatoblast-like cells and cholangiocyte-like cells.

### Cell composition of hepatic organoids is affected by local D2-mediated T3 production

**Figure 6:**
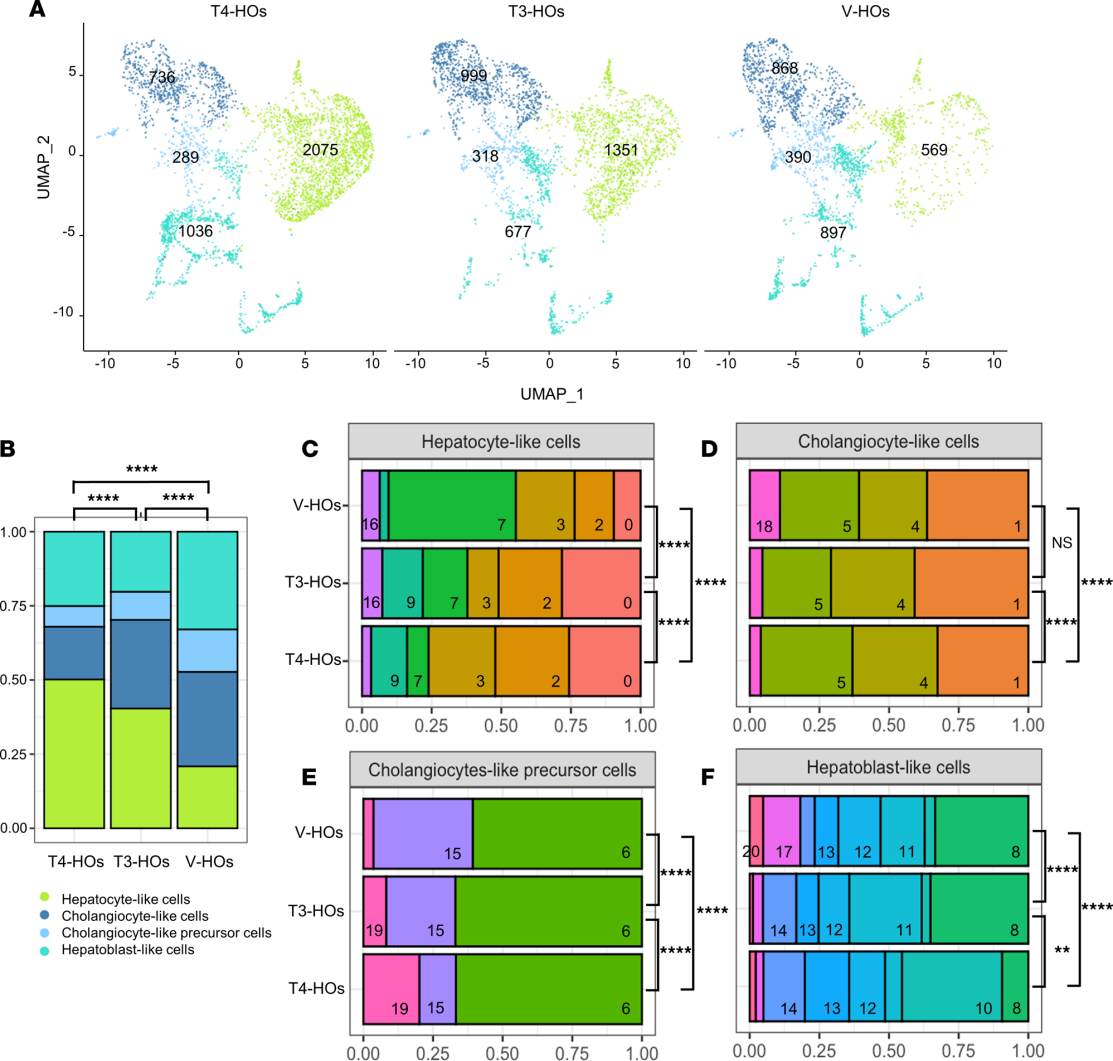
scRNA-Seq quantification of cells and gene expression in hepatic organoids at day 45. (**A**) UMAP visualization of V-HOs, T3-HOs, and T4-HOs. The number of cells is denoted in each group. (**B**) Histogram of the relative number of cells in groups treated with V-HOs, T3-HOs, and T4-HOs. (**C**–**F**) Histograms of the relative number of cells in clusters treated with V-HOs, T3-HOs, and T4-HOs. The identification number of each cell cluster is indicated at the bottom right corner of each rectangle. V-HOs (vehicle), T3-HOs (free T3 = ~10 pM), T4-HOs (free T4 = ~15 pM). The χ^2^ test for multiple comparisons and pairwise cell proportion. **P < 0.01; ****P < 0.0001.

Local D2-mediated T3 production influenced the hepatoblast maturation process, greatly affecting the proportion of the cell clusters (χ^2^ test, *P* < 0.0001) (Figure 6, A and B). The presence of either a functional D2-T3 pathway (χ^2^ test, *P* < 0.0001) or, to a lesser extent, T3 (χ^2^ test, *P* < 0.0001) was a critical determinant of the fate of the hepatoblasts (Figure 6, A and B). Within hepatocyte-like cells, all cell clusters were affected by TH signaling (χ^2^ test, *P* < 0.0001, Figure 6C). The presence of TH, with D2-T3 (χ^2^ test, *P* < 0.0001) or T3 (χ^2^ test, *P* < 0.0001), influenced the hepatocyte-like cell proportion. As an example, cluster 7 was the largest in V-HOs but dropped to one of the smallest with the addition of TH to the medium (Figure 6C).

TH effects were also present in cholangiocyte-like cells (χ^2^ test, *P* < 0.0001) and cholangiocyte-like precursor cells (χ^2^ test, *P* < 0.0001), but they were relatively less dramatic (Figure 6, D and E). Cholangiocyte-like cell clusters were similarly affected by T4 (χ^2^ test, *P* < 0.0001) but not by T3 (χ^2^ test, *P* < 0.093) (except for cluster 18, which responded prominently to TH; Figure 6D). Cholangiocyte-like precursor cells were relatively more responsive to TH, with clusters 19 (which expresses *THRB*; Supplemental Table 2) and 15 responding to TH (Figure 6E). As in hepatocyte-like cells, the intensity of the D2-T3 effects on the proportion of cells was greater than with T3 (χ^2^ test, *P* < 0.0001). As precursor cells, the hepatoblast-like cell clusters indicated a high response to TH (χ^2^ test, *P* < 0.0001). D2-T3 (χ^2^ test, *P* < 0.0001) and T3 (χ^2^ test, *P* < 0.0001) actions dramatically affected the hepatoblast-like cell clusters, except for clusters 12 and 13 (Figure 6F).

### Effects of local D2-mediated T3 production on the organoid transcriptome

Local D2-T3 production also markedly affected the organoids’ gene expression, as shown in the volcano plot distribution (Supplemental Figure 2, B–E) and in the cellular heatmaps (Supplemental Figures 3–5). The hepatocyte-like cell group was the most affected by D2-T3 (Supplemental Figure 2B), which modified the expression of 361 genes (302 were >1.5-fold upregulated). The changes in gene expression with T3 were less intense but qualitatively similar (Supplemental Figure 2B). As a group, these genes were involved in metabolic and developmental processes (Supplemental Table 3). The main pathways affected by D2-T3 included lipoprotein transport (fold-enrichment [FE]: 21), localization (FE: 20), alcohol metabolism (FE: 17), cholesterol metabolism (FE: 17), and sterol metabolism (FE: 17) (Supplemental Table 3). Other specific biological pathways were related to the estrogen metabolism and to the cholesterol and sterol homeostasis, with FE values of 11, 7.2, and 7.1, respectively (Supplemental Table 3). Of note, 71 differentially expressed genes (DEGs) (χ^2^ test, *P* < 0.00001) in the hepatocytes lacking D2-T3 (mainly involved in lipid metabolism) overlapped with the group of hepatic genes differentially expressed after the *Dio2* knockdown, confirming the relevance of these findings in organoids (Supplemental Figure 2F).

Hepatoblast-like cells were also sensitive to D2-T3, and this sensitivity affected the expression of 209 genes (121 were >1.5-fold upregulated); similarly to hepatocyte-like cells, incubation with T3 had qualitatively similar but less intense effects (Supplemental Figure 2C). Cholangiocyte-like cells and cholangiocyte-like precursor cells were much less sensitive to incubation with TH (Supplemental Figure 2, D and E). D2-T3 modified the expression of only 28 genes (18 were >1.5-fold upregulated), whereas, in cholangiocyte-like precursor cells, that number was 74 (55 were >1.5-fold upregulated). The effects of T3 were less substantial but qualitatively similar (Supplemental Figure 2, D and E).

## Discussion

In the present investigation, we studied the differentiation of human iPSCs into hepatic organoids and discovered a critical role played by local D2-mediated T3 production in the formation of hepatocyte-like cells. During day 14 to day 38, the hepatoblasts exhibited a marked induction of *DIO2* that was associated with accelerated local activation of T4 to T3. This period of enhanced T3 signaling spans from early hepatoblasts to hepatic organoids, which contain much more mature cells. The peak of *DIO2* expression overlapped with the developmental bifurcation of hepatoblasts to either hepatocyte- or cholangiocyte-like cells. In studies that used scRNA-Seq, we observed that eliminating the local D2-T3 signaling significantly affected the composition of mature cells and their transcriptome (day 45). In the absence of D2-T3 signaling, the number of hepatocyte-like cells decreased by ~60%, and the cholangiocyte-like cells increased by ~55%. The albumin and apolipoprotein content in the medium and mRNA levels of *ALB* and *KRT7* reflected these changes in cell number. Physiological levels of T3 were not able to fully normalize the transition from hepatoblasts to mature cells, indicating that the timed surge in D2-T3 signaling is a critical determinant of the fate of developing human hepatoblasts.

The timing of the D2-T3 signaling suggests that its main target is the hepatoblasts, potentially affecting the transition to mature cells. The induction of *DIO2* at this moment can be linked to the activation of the cAMP pathway, which, along with BMP, WNT, and FGF signaling, promotes hepatic specification in the organoids (17). Despite continued cAMP activation, *DIO2* induction subsides with the surge in the number of hepatocytes. These cells express liver X receptor, a known inhibitor of *DIO2* expression, which could be playing a role (27, 28).

Here, we saw that hepatoblasts are equipped to respond to D2-T3 given that, simultaneously with the increase in *DIO2* expression (day 14), there was an increase in *THRA* mRNA levels that persisted until day 46 (*THRB* mRNA are only detectable by day 29). The idea that the hepatoblasts are the D2-T3 targets is supported by the analysis of TF families that participate in and regulate the hepatoblast transition to mature cells. In the absence of D2-T3 signaling, there was a reduction in the expression of the 2 pioneer TFs *GATA4* and *FOXA1*; in the hepatoblast-specific TF *PROX1*; in the hepatocyte-promoting TFs *HNF4A* and *HNF1A*; and a reciprocal elevation in *HHEX*, a TF involved in bile duct morphogenesis.

Accordingly, *DIO2* is not expressed in mature hepatocytes or cholangiocytes; thus, it makes sense that, by day 38, *DIO2* mRNA levels were close to being undetectable. It is therefore likely that the dramatic changes in the cell composition observed in the developing hepatoblasts were the result of the D2-T3 acting in the expression of key TFs (*HNF4A*, *HNF1A*, and *CEBPA*) favoring the hepatocyte over the cholangiocyte-like lineage.

Nonetheless, studies based on TRβ1-KO iPSC–derived 2D monolayer cultures led to the idea that T3 accelerates hepatocyte proliferation (through TRβ) but not differentiation (29). However, here we found that hepatoblasts express *THRA*, which could mediate the effects of T3 on cell differentiation in the TRb1-KO monolayers. We also found that hepatocytes express high levels of *THRB* (but not the cholangiocyte-like cells) and, thus, are equipped to potentially respond to D2-T3. However, the timing of *DIO2* expression does not coincide with the increase in *THRB* expression, which started to increase by day 29. Thus, it is more likely that the pulse of D2-generated T3 in the developing hepatoblasts signals primarily via *THRA*. Although it has been reported that T3 added to the medium accelerates hepatocyte proliferation, here the growth curves and the final size of the organoids, as well as the levels of *MKI67* mRNA, were not affected by D2-T3, making it unlikely that hepatocyte proliferation is affected by the physiological D2-T3 pathway. Notably, at least some of the studies in 2D monolayers showing that T3 modifies the expression of liver-specific TFs (30) and known T3-target genes (31) were conducted with pharmacological T3 levels at 3–5 orders of magnitude higher than the physiological levels used in the present investigation.

In mice, the genetic inactivation of the *Dio2* pathway led to major changes in the transcriptome of the adult liver, and these changes were associated with a dramatic metabolic phenotype (15). *Dio2* expression occurs while important epigenetic reprogramming of the developing liver is taking place, and the transcriptome changes in the liver of mice with *Dio2* knockdown were linked to increased DNA methylation and reduced chromatin accessibility (15). Here, too, we observed that the hepatocyte-like cells generated from hepatoblasts lacking the D2-T3 pathway exhibited a markedly different transcriptome, even though the peak of *Dio2* expression occurred in the hepatoblasts. Hundreds of developmental and metabolic genes were differentially expressed in these hepatocyte-like cells that developed in the absence of D2-T3, including genes related to lipoprotein transport, alcohol metabolism, cholesterol metabolism, and sterol metabolism, reminiscent of what we saw in the mice with liver-specific *Dio2* inactivation (4, 15). Notably, 71 DEGs overlapped between the organoids lacking the D2-T3 pathway and the mice with liver-specific *Dio2* inactivation. This suggests that the epigenetic changes caused by D2-T3 occur early during hepatoblast differentiation.

Hepatic organoids mimic the structure interactions and microenvironment of the tissue (32, 33). While the utilization of these structures helps explain the developing human liver and can be used as a tool for potential disease treatments (34, 35), the relative immaturity of the fully differentiated organoids seen in the present investigation prevents us from concluding what the phenotype of the human liver could be in the absence of the *DIO2* pathway. This is illustrated by the presence of *AFP* mRNA in mature organoids (AFP levels are high in the blood of newborns but decrease to low values after 2 years old) (36). Despite the stepwise differentiation exhibited by these cells, the 3D hepatic organoid creates a reagent gradient between the outer and innermost cells, generating an undefined heterogeneity in the developmental process. This might explain the substantial presence of immature cells and uninterrupted expression of fetal markers in day 45 organoids.

The timed induction of *DIO2* in human liver organoids is reminiscent of what takes place in the brown adipose tissue (8), embryonic mouse liver (4), cochlea (10), and other tissues. It is known that plasma T3 levels in embryonic mammalian life are relatively low, and the timed exposure of developing tissues to T3 is controlled by coordinated peaks of *Dio2* (which produces T3) and troughs of *Dio3* (which inactivates T3 and T4). In the absence of this developmental pulse of T3 in the cochlea or brown adipose tissue, mice become deaf (37) or will exhibit impaired adaptive thermogenesis (38), respectively.

In conclusion, here we identified a peak of *DIO2* expression in the developing human hepatic organoid, and this peak is critical to defining the fate of developing hepatoblasts, favoring the hepatocyte lineage of cells. In the mouse model, the embryonic *Dio2* peak results in adult hepatocytes with reduced capacity to secrete VLDL, thus increasing susceptibility to liver steatosis in response to a high-fat diet or alcohol consumption (14). Indeed, patients with congenital hypothyroidism were found to have an increased risk of NAFLD and increased fasting glucose and insulin levels (39). Therefore, because of the similarities between the mouse (4, 15) and the organoid models, the present results likely carry significant physiological and clinical implications for how the liver regulates energy substrates and reacts to damage associated with high-fat diet and alcohol consumption.

## Methods

### iPSC maintenance.

Human iPSCs were obtained from the Cedars-Sinai Core (CS03iCTR-n3) and cultured on 6 well-plates (coated with Matrigel; Corning), using serum-free mTeSR+ (Stem Cell Technologies) medium. iPSC culture plates were at 37°C and 5% CO_2_. Divergence among the number of passages is 3 or lower.

### Hepatic organoids.

Differentiation used the serum-free reagent cocktails (C1–C6) previously described in ref. 40. In summary, day 0 iPSCs were treated with STEMdiff Definitive Endoderm Kit (Stem Cell Technologies) (C1) containing 50 μM Y-27632 (Tocris) to form differentiated into DE. Day 4 cells were then treated with 1× Glutamax supplement (Thermo Fisher Scientific), 1× B27 supplement (Thermo Fisher Scientific), 20 ng/mL BMP4 (R&D Systems), and 10 ng/mL FGF2 (R&D Systems) in Advanced DMEM/F12 (C2) for 6 days to form the PFG cells. Subsequently, day 10 cells were lifted using TrypLE Express Enzyme (Thermo Fisher Scientific) and split into 96-well ultra-low attachment (ULA) plates (Corning) at ~30,000 cells/well in medium —advanced DMEM/F12, including 1× N2 supplement (Thermo Fisher Scientific), 1× B27, 50 nM A83-01 (Tocris), 30 μM dexamethasone (Tocris), 5 μM CHIR99021 (Tocris), 500 nM valproic acid (R&D Systems), 50 ng/mL human epidermal growth factor (EGF) (R&D Systems), 20 ng/mL human hepatocyte growth factor (HGF) (R&D Systems), 40 ng/mL Jagged-1 (R&D Systems), 300 ng/mL dbCAMP (Sigma-Aldrich), and 10 μM nicotinamide (Tocris) — containing 1× Matrigel for 8 days, to promote reorganization into a hepatic intermediate stage. Organoids were then gathered in 6-well ULA plates (10 organoids per well) kept on a multiplatform shaker (Thermo Fisher Scientific) at 65 rpm, in a modified C3 medium — valproic acid was removed and 25 ng/mL FGF7 (R&D Systems), 50 ng/mL BMP4, and 20 ng/mL BMP7 (R&D Systems) were incorporated (C4) — for the next 8 days. For the hepatic organoid maturation, we used the same reagents as in C4, reducing to 2 μM CHIR99021 and 20 ng/mL BMP4 and removing Jagged-1 (C5). Following 12 days, BMP4, BMP7, and FGF7 were substituted by 25 ng/mL FGF19 (R&D Systems), and 5 μM DAPT (Tocris) for another 8 more days (C6). The size of the organoids was measured using images captured by the Infinity1 (Teledyne Lumenera) camera and the Infinity Analyze software.

### B26 constitution for V-HOs, T4-HOs, and T3-Hos.

The concentration of T3 in the B27 medium is 2.6 nM (24). For the B26 constitution, we followed the descriptive steps in ref. 41, with the exception of adding T3 to the mix. For the experiments in which V-HOs, T4-HOs, and T3-HOs were used, the B26-reagent cocktail was supplemented with L-thyroxine (T4, Sigma-Aldrich), 3,3’,5-triiodo-L-thyronine (T3, Sigma-Aldrich), or vehicle and used at the indicated times.

### Immunofluorescence.

Cells or organoids taken for immunostaining were washed with PBS and fixed with 4% paraformaldehyde for 15 minutes at room temperature (RT), blocked with 2.5% normal horse serum (Vector Laboratories) for 20 minutes, washed with PBS, and incubated with the primary antibody (Supplemental Table 4) in PBS-Triton 0.5× overnight at 4°C. As indicated, 2 primary antibodies were used during the same incubation only if they were prepared in different species as described in ref. 42. Cells and organoids were then washed with PBS, and the appropriate secondary antibodies (Supplemental Table 4) were added for 2 hours at RT. Nuclei were visualized with DAPI. Images were obtained with a Nikon Confocal D-Eclipse C1si Laser Scanning Confocal microscope. After taking the pictures, the background was reduced and, when appropriate, the channels (colors) were split and individual colors balanced using Adobe Photoshop.

### Quantitative PCR (qPCR) and ELISA.

RNA from hepatic organoids was extracted using RNAqueous-Micro Total RNA Isolation Kit (Invitrogen) and, in 2D stages, RNeasy Mini Kit (Qiagen) was used. The RNA amount and quality were analyzed in DeNovIx DS-11 Spectrophotometer. RNA was retrotranscribed into complementary DNA (cDNA) using Transcriptor First Strand cDNA Synthesis Kit (Roche) in Mastercycler nexus – PCR Thermal Cycler. PowerUp SYBR Green Master Mix (Applied Biosystems) probes were used to detect gene expression in StepOnePlus Real-Time PCR System by qPCR. β-Actin was used as the internal control, and the cycle threshold was compared with the relative standard curve for analysis. Albumin, apolipoprotein B, and apolipoprotein A1 concentration in the medium was measured using human albumin ELISA kit (Bethyl Laboratories), Human Apolipoprotein B/ApoB Quantikine ELISA Kit (R&D systems), and Human Apolipoprotein A/ApoA1 Quantikine ELISA Kit (R&D systems), respectively.

### Deiodinase assay.

Four organoids per time-point were sonicated in PE (DPBS [Thermo Fisher Scientific], 1 mM EDTA [Thermo Fisher Scientific]), 0.25M Sucrose (MilliporeSigma), and 20 mM DTT (MilliporeSigma) (3 pulses of 2 seconds at 40% amplitude in Qsonica Q125). Protein concentration was measured using Protein Assay Dye Reagent Concentrate (Bio-Rad). D2 and D3 assays were performed at day 22 and day 38. [^125^I-T4] and [^125^I-T3] were purified through Sephadex (MilliporeSigma) columns. For the D2 assay, 50 μL of the sample with 250 μL of the reaction mix (1 mM 6-propyl-2-thiouracil [PTU; MilliporeSigma], 20 mM DTT, 10 nM T3 [MilliporeSigma], 0.5 nM T4 [MilliporeSigma], and 2 × 10^5^ counts per minute (cpm) [^125^I-T4] per reaction) at 37°C for 3 hours. Protein was precipitated with 200 μL of horse serum (Thermo Fisher Scientific) and 100 μL 50% Trichloroacetic acid (Thermo Fisher Scientific), vortexed for 2 minutes, centrifuged for 5 minutes at 21,130 rfc at room temperature for 5 minutes, and measured for free ^125^I in 2470 Automatic Gamma Counter Wizard^2^ (Perkin-Elmer). For the D3 assay, 60 μL of the sample was combined with 40 μL of the reaction mix (1 mM PTU, 20 mM DTT, 0.5 nM T3, 10 nM T4, and 1.2 × 10^5^ cpm [^125^I-T3] per reaction) at 37°C for 3 hours and 30 minutes. We added 100 μL MetOH (Thermo Fisher Scientific), and it was centrifuged for 5 minutes at 21,130 rfc at room temperature for 5 minutes for precipitation. We took 150 μL of the supernatant, added 130 μL HPLC mix, passed it through 0.45 μm Centrifuge Tube Filter (Corning), and analyzed it via Acquity UPLC class (Waters).

### scRNA-Seq.

The reagents were prewarmed at 37°C. Six organoids (day 45) were recollected in a 15 mL polypropylene (Falcon) tube to wash with PBS 3 times. As a dissociation reagent, 1 mL of trypsin 0.5% — EDTA (10×) (Thermo Fisher Scientific) with 50 μM Y-27632 (Thermo Fisher Scientific), 40 U/μL RNase inhibitor (Invitrogen), and 1 U DNase I (Thermo Scientific) was added to the organoids and kept on 37°C in a Vari-Mix Platform Rocker (Thermo Fisher Scientific) at 30 rpm for 15 minutes. Every 5 minutes, we mechanically disrupted the organoids until it was homogenized into single cells. Afterward, we proceeded to the inactivation of trypsin with 1 mL Advanced DMEM/F12 mix containing Bovine Albumin Fraction V (7.5% solution, Thermo Fisher Scientific) 50 μM Y-27632 (Thermo Fisher Scientific), 40 U/μL RNase inhibitor (Invitrogen), and 1U DNase I (Thermo Fisher Scientific). We collected and filtered the 2 mL through a 40 μm Cell Strainer (Corning) into a 50 mL tube (Falcon). We then add another 1 mL Advanced DMEM/F12 mix to recover any left cells from the strainer. We took 10 μL of single-cell medium mixed with 10 μL Trypan Blue (0.4%) to count the number of single cells to proceed with their fixation. We carried out the fixation of at least 1 × 10^5^ required single cells, following the protocol described in the Evercode fixation kit (Parse Bioscience). The preparation for the cell barcoding and the posterior library for sequencing was performed with Evercode WT Mini v2 (Parse Bioscience). The Genomics Core facility of the University of Chicago performed the sequencing.

### Single-cell RNA-Seq analysis.

The libraries were prepared using the Parse Bioscience kit and were sequenced using a HiSeq Illumina platform, paired-end setting. The produced fastq files were analyzed with the Parse Bioscience pipeline suite 1.0.4. The suite performed reads demultiplexing and aligned the reads against a STAR-produced index reference from the Ensembl human whole genome reference GRCh38.108, followed by the calculation of read counts per feature per cell. The suite output was analyzed using Seurat (43). The library selected for the downstream analysis included data from 10,887 cells. For quality control (QC), we set cells with, at most, 9,000 features, 61,000 cumulative counts, and less than 10% of mitochondrial DNA. These cutoffs corresponded to the 95% quantiles for the 3 metrics and were set to discard cells with low transcripts or multiplets. All the cells had more than 2,921 features with nonzero counts and more than 6,733 reads. After QC analysis and filtering, we selected genes with the most significant variance, filtering to maintain 3,000 genes with a standardized variance of >1.96. The resultant matrix was used for principal component analysis (PCA) for dimension reduction. We ran nonlinear dimensional reduction by Uniform Manifold Approximation and Projection (UMAP) and clustering by the shared nearest neighbor (SSN) approach at 0.8 resolution using the first 20 principal components corresponding to 80% of the data variation. Cell annotation was done empirically, informed by computed conserved gene markers for each cell cluster and by median expression of known cell markers. As a result, we separated the 21 clusters into 4 cell types. The differential expression analysis was computed using the DeSeq2 and MAST methods in the Seurat environment. We used *P* < 0.01 and fold change >1.5 as thresholds for DEGs, comparing each treatment regimen against the vehicle group as the control.

### Statistics.

Data are presented as mean ± SD and are aligned or in scatter dot plots. As appropriate, groups were compared using a 2-tailed Student’s *t* test, 1-way ANOVA, and a Tukey and Bonferroni post hoc test. GraphPad Prism 9 software was used for the statistical analysis. For scRNA-Seq, we performed χ^2^ tests to identify differences in the proportions of cells between treatment regimes. A threshold for a *P* value less than 0.05 was considered significant.

### Data availability.

The Supporting Data Values file reports the data from Figures 1–4 and Supplemental Figure 1. Single-cell RNA-Seq (day 45) data are available in the repository of Gene Expression Omnibus (GEO) belonging to the National Center for Biotechnology Information (NCBI) (no. GSE245250).

## Author contributions

JHA performed all experiments and data analysis and wrote and prepared the manuscript, figures, and tables; JHA and FSL planned the organoid protocol; JHA and TLF performed the fixation and libraries for scRNA-Seq; DVC carried out the scRNA-Seq downstream analysis; and ACB planned and directed all studies and the manuscript write-up.

## Supplementary Material

Supplemental tables 1-2

Supplemental table 3

Supplemental table 4

Supporting data values

## Version 1 10/19/2023

In-Press Preview

## Version 2 12/08/2023

Electronic publication
