# Supplementary material for: Localized T3 production modifies the transcriptome and promotes the hepatocyte-like lineage in iPSC-derived hepatic organoids
Source: JCI Insight. 2023 Dec 8;8(23):e173780. doi: 10.1172/jci.insight.173780 (PMC10795825; doi:10.1172/jci.insight.173780)
Supplement: Supplemental tables 1-2 [file jciinsight-8-173780-s007.pdf]

Tables

Table 1. Forward and Reverse sequences of primers for the quantitative polymerase chain reaction (qPCR) in human hepatic organoids.

Gene	5'– Forward – 3'	5'– Reverse – 3'
hAFP	CCAGGAACAGGAAGTCTGCT	CACACCGAATGAAAGACTCG
hALB	TGCTGAGGCAAAGGATGTCT	ATGTCTTGGCAAGTCTCAGCA
hB-ACTIN	GTGGACATCCGCAAAGACCT	ACTTGCGCTCAGGAGGAGC
hCDX2	CCGCAGAGCAAAGGAGAGGA	TGCAGGGAAGACACCGGACT
hCEBPA	AGCCTTGTTTGTACTGTATG	AAAATGGTGGTTTAGCAGAG
hCER1	TGGCACCACTTCATGTTCAGAAA	TGAGAGCAGGAGGTATGGGAGTG
hCYP3A4	GTAAAAAGGATGAAAGAAAGTCGCC	TCATCAGCTGAAGGAAATCCACT
hCYP3A7	CGTAAGTGGAGCCTGATTTCCCT	AATGGTGCTAACTGGGGGTGGTG
hDIO1	GTCGTGGGTAAAGTGCTTCTG	GTTCCGCTTGACTCTGTCTGG
hDIO2	GCTGCTGTTGAGCCGCTC	GCTCAGGGCTGGCAAAGTC
hDIO3	GCTGGTTCTCAATTTCGGCA	GCTGTGGGATGATGTAGGGA
hFOXA1	GCAATACTCGCCTTACGGCT	TACACACCTTGGTAGTACGCC
hFOXA2	GGAACACCACTACGCCTTCAAC	AGTGCATCACCTGTTCGTAGGC
hGATA4	CGACACCCCAATCTCGATATG	GTTGCACAGATAGTGACCCGT
hHHEX	ACGCCCTTTTACATCGAGGAC	CGTGTAGTCGTTCACCGTC
hHNF1A	AACACCTCAACAAGGGCACTC	CCCCACTTGAAACGGTTCCT
hHNF1B	GTGGACCGGATGCTCAGTG	GGGTCTTCATAGGGGTGCC
hHNF4A	TGCAGGTGTTGACGATGGGCA	ACCACGCACTGCCGGCTAAAT
hKRT7(CK7)	AGCCGTGAATATCTCTGTGATGAA	AATAAGCCTTCAGGAGCCCAG
hMKI67	TGACCCTGATGAGAAAGCTCAA	CCCTGAGCAACACTGTCTTTT
hNCOA1(SRC1)	CTCGGGGACAGTTCATCCG	CCGTGCTTGATGCCAGTGT
hNCOR1	ACACCGCAGTATTGTCCAAAT	CACCTGGTTTGTCTTGATGTTCT
hNCOR2	TGCAGATCATCTACGACGAGA	TCCGCATCGCCTGGTTTATTT
hONECUT1(HNF6)	AGCGTCGAACTCTACATGCAA	TGCTTTGGTACAAGTGCTTGAT
hOTX2	ACCCGGTACCCAGACATCTTCAT	GGCCACTTGTTCCACTCTCTGAA
hPOU5F1(OCT4)	CTTCGCAAGCCCTCATTTCACCA	GCACTAGCCCCACTCCAACCTG
hPROX1	AAAGGACGGTAGGGACAGCAT	CCTTGGGGATTCATGGCACTAA
hSOX2	CCCAGCAGACTTCACATGT	CCTCCCATTTCCCTCGTTTT
hTBX3	AAAAATAGACAACAACCCTTTTGC	ACTGCAGGGTGAGCTGTTTT
hTHRA	AAGGCAACTGGTTATCACTACCG	ACGGCGATGCACTTCTTGAA
hTHRB	TTCCAAACGGAGGAGAAGAA	TAGTGATACCCGGTGGCTTT

Table 2. Antibodies used for immunofluorescence of the hepatic organoids.

**Antibody**	**Concentration**	**Brand**	**Catalog #**
Anti-hOTX2	1:250	Novus	AF1979
HNF4α Monoclonal Antibody	1:250	Invitrogen	MA1-199
Anti-TBX3 antibody	1:250	Abcam	AB99302
Human Albumin Polyclonal Antibody	1:250	Bethyl	A80-129A
Ms x Hu KI-67	1:250	Sigma Aldrich	MAB4190
Anti-Cytokeratin 7 antibody	1:250	Abcam	AB53123
Duet Immunofluorescence Double Labeling Kit, DyLight™ 594 Anti-Rabbit, DyLight™ 488 Anti-Mouse	Ready-to-use	VectaFluor™	DK-8828
Anti-Rabbit IgG, DyLight™ 488	Ready-to-use	VectaFluor™	DI-8828
Horse Anti-Goat IgG (H+L), DyLight™ 594	1:250	VectaFluor™	DI-3094
Horse Anti-Mouse IgG (H+L), DyLight™ 488	1:250	VectaFluor™	DI-2488
